# Distinct roles of immunoreceptor tyrosine‐based motifs in immunosuppressive indoleamine 2,3‐dioxygenase 1

**DOI:** 10.1111/jcmm.12954

**Published:** 2016-09-30

**Authors:** Elisa Albini, Verdiana Rosini, Marco Gargaro, Giada Mondanelli, Maria L. Belladonna, Maria Teresa Pallotta, Claudia Volpi, Francesca Fallarino, Antonio Macchiarulo, Cinzia Antognelli, Roberta Bianchi, Carmine Vacca, Paolo Puccetti, Ursula Grohmann, Ciriana Orabona

**Affiliations:** ^1^ Department of Experimental MedicineUniversity of PerugiaPerugiaItaly; ^2^ Department of Pharmaceutical SciencesUniversity of PerugiaPerugiaItaly

**Keywords:** IDO1, immunoreceptor tyrosine‐based inhibitory motifs (ITIMs), immunosuppression, protein phosphorylation, proteasome, plasmacytoid dendritic cell, tyrosine phosphatase, non‐canonical NF‐κB, phosphotyrosine

## Abstract

The enzyme indoleamine 2,3‐dioxygenase 1 (IDO1) catalyses the initial, rate‐limiting step in tryptophan (Trp) degradation, resulting in tryptophan starvation and the production of immunoregulatory kynurenines. IDO1's catalytic function has long been considered as the one mechanism responsible for IDO1‐dependent immune suppression by dendritic cells (DCs), which are master regulators of the balance between immunity and tolerance. However, IDO1 also harbours immunoreceptor tyrosine‐based inhibitory motifs, (ITIM1 and ITIM2), that, once phosphorylated, bind protein tyrosine phosphatases, (SHP‐1 and SHP‐2), and thus trigger an immunoregulatory signalling in DCs. This mechanism leads to sustained IDO1 expression, in a feedforward loop, which is particularly important in restraining autoimmunity and chronic inflammation. Yet, under specific conditions requiring that early and protective inflammation be unrelieved, tyrosine‐phosphorylated ITIMs will instead bind the suppressor of cytokine signalling 3 (SOCS3), which drives IDO1 proteasomal degradation and shortens the enzyme half‐life. To dissect any differential roles of the two IDO1's ITIMs, we generated protein mutants by replacing one or both ITIM‐associated tyrosines with phospho‐mimicking glutamic acid residues. Although all mutants lost their enzymic activity, the ITIM1 – but not ITIM2 mutant – did bind SHPs and conferred immunosuppressive effects on DCs, making cells capable of restraining an antigen‐specific response *in vivo*. Conversely, the ITIM2 mutant would preferentially bind SOCS3, and IDO1's degradation was accelerated. Thus, it is the selective phosphorylation of either ITIM that controls the duration of IDO1 expression and function, in that it dictates whether enhanced tolerogenic signalling or shutdown of IDO1‐dependent events will occur in a local microenvironment.

## Introduction

IDO1 is a metabolic enzyme responsible for degrading the essential amino acid l‐tryptophan (Trp) into l‐kynurenine (Kyn), which represents the upstream metabolite along the so‐called kynurenine pathway 1, 2. Over the years, this ancestral enzyme has proven to be a powerful immunoregulator that, by using Trp depletion and/or Kyn production, presides over immune homoeostasis in adult life 3, 4, 5, 6. In addition to its metabolic activity, a signalling function has recently been described for the phosphorylated form of the IDO1 protein (pIDO1), owing to the identification of two highly conserved amino acid sequences – namely, ITIM1 and ITIM2 – containing a phosphorylable tyrosine residue 7, 8, 9.

Much like ITIMs in several inhibitory immunoreceptors 10, 11, 12, phosphorylated ITIMs in IDO1 act as docking sites in the association with different molecular partners, leading to disparate outcome. We initially identified SOCS3 as one protein capable of anchoring pIDO1 through its Src homology 2 (SH2) domain. In an interleukin 6 (IL‐6)–rich inflammatory milieu 7, IL‐6‐induced SOCS3 will recruit E3 ubiquitin ligase complex which, in turn, promotes ubiquitination and proteasomal degradation of the enzyme. Later in time and in a different setting, we found that pIDO1 can likewise bind SHP‐1 and SHP‐2 tyrosine phosphatases, resulting in signalling ability of the pIDO1 molecule. Besides the absence of IL‐6, crucial to the signalling events initiated by pIDO1 is the cytokine transforming growth factor β (TGF‐β) 8. Thus, in a TGF‐β‐dominated environment, pIDO1 interacts with SHPs to enhance its own transcription as well as that of TGF‐β, in a feedforward loop that sustains an anti‐inflammatory programme in plasmacytoid dendritic cells (pDCs), which are master regulators of immune homoeostasis 8, 13.

We here examined the specific contribution of each ITIM to the association of pIDO1 with either type of protein partner (*i.e*. SOCS3 *versus* SHPs). By using murine IDO1 mutants mimicking stable and specific tyrosine phosphorylation of ITIM1, ITIM2, or both, we identified distinct roles for ITIM1 and ITIM2 in the selective association with their protein partners and the resulting opposite outcome. The identification of mechanisms that diametrically control the functions and fate of IDO1 opens up new perspectives for pharmacologic control of IDO1‐related events.

## Materials and methods

### Mice, cell lines, and reagents

Female wild‐type C57BL/6 mice were purchased from Charles River Breeding Laboratories (Calco) and female IDO1‐deficient (*Ido1* ^*−*^ */* ^*−*^) C57BL/6 mice were purchased from Jackson Laboratories and bred at Charles River Breeding Laboratories. All animals were housed and fed under specific pathogen‐free conditions. All *in vivo* studies were in compliance with National (Italian Animal Welfare Assurance A‐3143‐01) and Perugia University Animal Care and Use Committee guidelines. P1.HTR, a highly transfectable clonal variant of mouse mastocytoma P815 14, was cultured in Iscove's modified Dulbecco's medium supplemented with 10% FCS. The proteasome inhibitor, MG132, was purchased from Calbiochem (Darmstadt, Germany). The *H‐2D* ^b^‐restricted HY peptide (WMHHNMDLI) – containing the immunodominant epitope of the male mouse‐specific minor transplantation antigen – was synthesized by BioFab Research (Rome, Italy).

### Molecular electrostatic potential (MEP) analysis

*In silico* models of l‐glutamate (l‐Glu), l‐tyrosine (l‐Tyr) and l‐phosphotyrosine (l‐pTyr) were generated using Maestro (version 9.5), as implemented in Schrödinger software modules. l‐Glu and l‐pTyr were considered as bearing a formal charge of −1. Geometry optimizations were carried out using quantum mechanics methodology with density functional theory (DFT) B3LYP/6‐31G** and diffuse++ function. All DFT calculations were carried out using Jaguar software (version 7.9), with a medium grid density and a fully analytic accuracy level. Atomic electrostatic potential charges (ESP) were calculated by fitting the electrostatic potential on atom centres. Geometry optimizations were performed with the standard Poisson–Boltzmann solvent model. The electrostatic potential surface was generated by solving the Poisson–Boltzmann equations and using ESP charges of all atoms in the molecule. Values for each compound were mapped on the solvent accessible molecular surface of l‐Glu, l‐Tyr and l‐pTyr respectively.

### Solvent‐accessible surface area

Crystal structures of IDO1 were obtained from the Protein Data Bank 15. Those included 10 structures with the following pdb codes: 2D0T, 2D0U, 4PK5, 4PK6; 4U72, 4U74, 5EK2, 5EK3, 5EK4, 5ETW 16, 17, 18. The POPS program was used to calculate solvent‐accessible surface area (SASA) of residues and the fraction of SASA relative to total surface area of a residue (Q_SASA_) 19.

### Construction and expression of mouse *Ido1* wild‐type and mutants

Constructs expressing wild‐type (WT) *Ido1* were generated from cDNA of conventional dendritic cells (DCs) stimulated with IFN‐γ, as previously described 8. For *Ido1* amplification, primers containing *Spe*I (sense) and *Not*I (antisense) restriction sites were used (Table 1). *Ido1* mutants were obtained by PCR‐based site‐directed mutagenesis. Complementary and overlapping primers containing specific substitutions (Table 1) were used to generate two DNA fragments with overlapping ends, which were combined in a subsequent ‘fusion’ product – further amplified by PCR – incorporating the specific nucleotide substitution. PCR products were digested with appropriate restriction enzymes and cloned into the pEF‐BOS plasmid 20. For both WT and mutated *Ido1* constructs, 20 μg DNA was used to transfect 1 × 10^7^ P1.HTR cells by electroporation, and stable transfectants were obtained by puromycin selection. P1.HTR cells, stably transfected with the empty plasmid, were used as a control.

**Table 1:** Primer sequences used in this study

Primers	Sense and antisense oligonucleotides
WT	S 5′‐GGCCAA*ACTAGT*GGTCAGTGGAGTAGACAGCA‐3′
	AS 5′‐CTCTTCTGAGTTGGCCTTAGT*GCGGCCGC*CTCCACT‐3′
Y115E	S 5′‐TATTGCTGTTCCC*GAA*TGCGAGCTCTCAGAG‐3′
	AS 5′‐CTCTGAGAGCTCGCA*TTC*GGGAACAGCAATA‐3′
Y253E	S 5′‐AAGGTCTGCTG*GAA*GAGGGGGTCTGG‐3′
	AS 5′‐CCAGACCCCCTC*TTC*CAGCAGACCTT‐3′

Mutated nucleotides are indicated in italics.

### Real‐time PCR

Real‐time PCR analyses were performed as described 8 using primers specific for *Ido1* (sense 5′‐CGATGTTCGAAAGGTGCTGC‐3′; antisense 5′‐GCAGGAGAAGCTGCGATTTC‐3′) and *Gapdh* (sense 5′‐CTGCCCAGAACATCATCCCT‐3′; antisense 5′‐ACTTGGCAGGTTTCTCCAGG‐3′). Values were expressed as the ratio of *Gapdh*‐normalized transcript expression of *Ido1* mutated forms to *Gapdh*‐normalized transcript expression of WT *Ido1*.

### Immunoprecipitation and immunoblot analyses

Stably transfected cells – to be immunoblotted as such (whole‐cell lysate, WCL; 1 × 10^5^ cells/sample) or after immunoprecipitation (IP; 6 × 10^6^/sample) – were lysed on ice in RIPA buffer (50 mM Tris‐HCl, pH 7.4, 150 mM NaCl, 1% Nonidet P‐40, 0.25% Na‐deoxycholate, 1 mM EDTA, 1.4 mM Na_3_VO_4_ and protease inhibitors). Lysates were first immunoprecipitated by means of rabbit polyclonal antibody specifically recognizing murine IDO1, raised in our laboratory 21, and then incubated with protein G‐agarose (Sigma‐Aldrich, Missouri, USA) in IP experiments; alternatively, WCLs were run directly on SDS/PAGE.

Immunoblotting involved the use of specific antibodies (rabbit polyclonal antibody recognizing murine IDO1, raised in our laboratory; murine SOCS3 from Abcam and murine tyrosine phosphatase SHP‐1 and SHP‐2, from Santa Cruz Biotechnology, Dallas, Texas 75220 USA) in combination with appropriate horseradish peroxidase‐conjugated antibody (Millipore, Darmstadt, Germany), followed by enhanced chemiluminescence (ECL). Anti‐β‐tubulin (Sigma‐Aldrich) was used as a normalizer.

Densitometric analysis of specific signals was performed within a linear range of blot exposure, in each experiment selecting the two lowest‐exposure times required for detecting signals.

### Kynurenine and phosphatase assays

The enzymatic activity of IDO1 was measured *in vitro* in terms of the ability of stably transfected cells – incubated for 6 or 16 hr in a medium supplemented with 100 μM Trp – to metabolize Trp to Kyn, whose concentrations were measured by HPLC in culture supernatants. The detection limit of the assay was 0.05 μM. WT and mutated IDO1 proteins were immunoprecipitated and assayed by means of a phosphatase assay kit according to manufacturer's instructions (Sigma‐Aldrich).

### DC purification, transfections and gene silencing

All purification procedures of splenic *Ido1* ^*−/−*^ pDCs and CD8^−^ DCs (hereafter referred to as cDCs, for conventional DCs) were as described 7, 8, 22, 23, 24, 25. Plasmid constructs coding for WT or IDO1 mutants (Y115E, Y253E and Y115_253E, in which either one or both phosphorylable tyrosines were replaced by a glutamate residue) were used as templates for *in vitro* transcription of mRNA, using the mMESSAGE mMACHINE T7 Ultra Kit (Ambion, Waltham, MA USA). *In vitro* transcribed‐mRNA (2 μg) was transfected into *Ido1* ^*−/−*^ pDCs by means of DOTAP (Roche, Basel, Switzerland), as described 7, 13. Control treatments consisted of *Ido1* ^*−/−*^ pDCs transfected with a control mRNA supplied by the manufacturer (Ambion). For silencing *Ptpn6 and Ptpn11* (coding for SHP‐1 and SHP‐2 phosphatases respectively), gene‐specific small interfering RNA (siRNA) was designed on the basis of the target sequence and synthesized by Ambion Life Technologies, which also supplied the negative control siRNA (NC siRNA). A one‐to‐one mix of *Ptpn6‐ and Ptpn11‐*specific siRNA was transfected into *Ido1* ^*−/−*^ pDCs for gene silencing. Transfected cells were incubated at 37°C in medium alone and, after 24 hr, recovered, washed and immediately used for *in vitro* and *in vivo* experiments.

### Nuclear translocation of non‐canonical NF‐κB subunits and TGF‐β detection

Nuclear and cytoplasmic extracts from *Ido1* ^*−/−*^ pDCs, transfected with WT and ITIM‐mutated *Ido1* mRNA and incubated for 48 hr at 37°C, were prepared according the manufacturer's protocol for the TransAM Flexi NF‐κB Family Kit (Active Motif). Analysis of p52 and RelB expression was performed on normalized samples of pDCs using rabbit polyclonal anti‐p100/p52 (Cell Signaling Technology, MA, USA), anti‐RelB (Santa Cruz Biotechnology) antibodies. Anti–β‐tubulin and anti‐Lamin B1 (Thermo Fisher Scientific, Massachusetts, USA) were used as normalizers of the cytoplasmic and nuclear extracts respectively. TGF‐β production in culture supernatants from those cells was measured by means of TGF beta 1 Emax Immuno Assay System (Promega, Madison, WI 53711 USA).

### Immunization and skin test assay

A skin test assay was used for measurements of major histocompatibility complex class I‐restricted delayed‐type hypersensitivity (DTH) responses to the HY peptide 8, 25 in C57BL/6 female recipient mice. For *in vivo* immunization, 3 × 10^5^ peptide‐loaded cDCs, combined with a minority fraction (5%) of peptide‐loaded transfected *Ido1* ^*−/−*^ pDCs, were injected subcutaneously into recipient mice. Two weeks later, a DTH response was measured to intrafootpad challenge with the eliciting peptide, and results were expressed as the increase in footpad weight of peptide‐injected footpads over that of vehicle‐injected (internal control) counterparts 24.

### Statistical analysis

Student’*s t*‐test was used to analyse the results of *in vitro* studies in which data were mean values (±S.D.). All experiments were performed at least three times using triplicate samples. In the *in vivo* skin test assay, statistical analysis was performed with two‐tailed paired *t*‐test, by comparing the mean weight of experimental footpads with that of control, vehicle‐injected counterparts 26. Data are means (±S.D.) of three experiments with at least six mice per group per experiment, as computed by power analysis to yield a power of at least 80% with an α‐level of 0.05 8.

## Results

### Replacement of tyrosine with glutamate in IDO1 ITIMs by site‐directed mutagenesis

**Figure 1:**
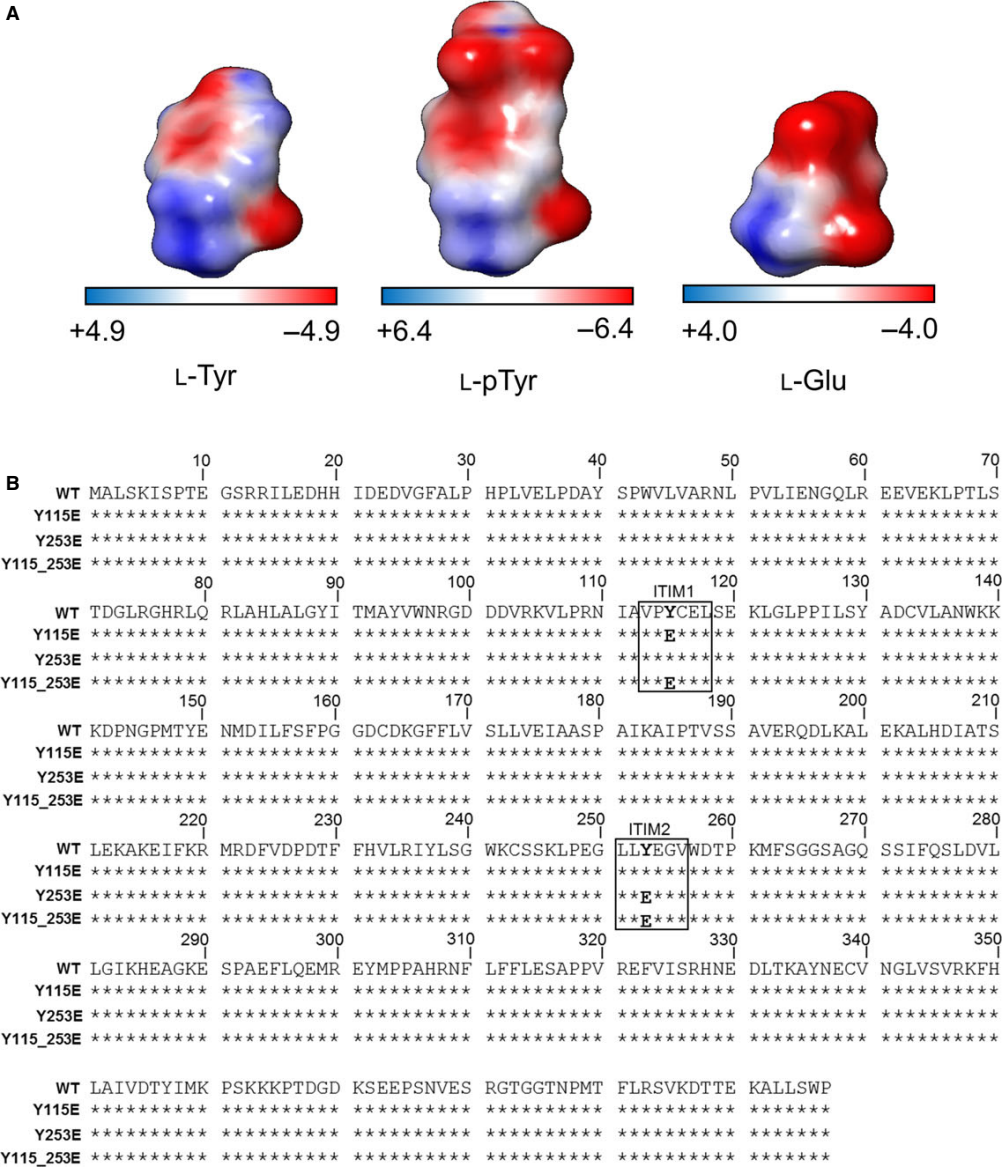
Replacement of tyrosine with glutamate in IDO1 ITIMs. (**A**) MEP analysis of l‐Tyr, l‐pTyr and l‐Glu. Red and blue coloured surfaces indicate electrostatic negative and positive potential regions respectively. (**B**) Peptide sequence alignment encoded by murine *Ido1* and its mutated forms as deduced by gene sequencing. Stars indicate residues identical to the WT sequence (NCBI Reference Sequence: NP_032350.1). ITIM1 and ITIM2 consensus sequences are shown in the inset and bold letters indicate the amino acid substitution.

Phosphorylation of the IDO1 protein occurs in DCs and pDCs, and it involves the tyrosine‐115 (Y115) and tyrosine‐253 (Y253) residues, present in the respective ITIM1 and ITIM2 motifs 7, 8. When phosphorylated, Y115 and Y253 acquire a negative net charge, likely affecting the conformational state and functional pattern of the enzyme 9. The new conformation, transiently acquired by pIDO1, can be mimicked by replacing ITIM tyrosines (Y) with glutamate (E) 27, 28, 29, which provides a negatively charged site similar to that of pY115 and pY253. Supporting this notion, MEP analysis showed a similar distribution of the electrostatic negative potential surface in l‐pTyr and l‐Glu amino acidic residues relative to the l‐Tyr neutral aromatic residue (Fig. 1A).

Gene constructs encoding the Y115E (ITIM1), Y253E (ITIM2) or Y115_253E (ITIM1 and 2) mutated forms of the enzyme were obtained by PCR‐based site‐directed mutagenesis from WT *Ido1*, by incorporating nucleotide changes for the substitution of either one or both phosphorylable ITIM tyrosines with a glutamate residue (Fig. 1B). WT IDO1 and mutants thereof were then used as a means of discriminating the effect of selective phosphorylation at either ITIM site in IDO1.

### Expression and catalytic activity of ITIM‐mutated IDO1 in transfected cells

**Figure 2:**
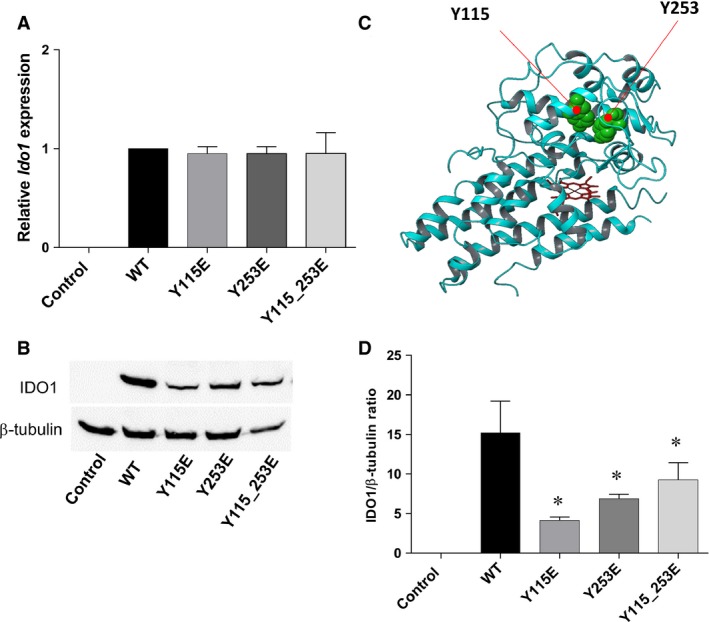
Expression of ITIM‐mutated IDO1 in transfected cells. (**A**) *Ido1* mRNA was quantified by real‐time PCR using *Gapdh* normalization. Data are presented as normalized transcript expression of the mutated forms of *Ido1* relative to normalized transcript expression of the WT *Ido1*. Results are representative of three experiments (mean ± S.D.). (**B**) and (**C**) IDO1 protein expression was analysed in WCL of WT and ITIM‐mutated IDO1 transfectants, using an IDO1‐specific antibody and anti‐β‐tubulin for normalization. One representative immunoblot analysis of three is shown. IDO1:β‐tubulin ratio was calculated by densitometric quantification of the specific bands detected in three independent experiments (mean ± S.D.). Controls in A, B and C are represented by mock‐transfected cells. *P < 0.05, mutants *versus* WT (Student’*s t*‐test). (**D**) Ribbon structure of IDO1 (pdb code: 2D0T) showing Y115 and Y253 (green balls) with poor solvent‐accessible surface.

ITIM‐mutated *Ido1* constructs were transfected into P1.HTR cells to obtain stable cell lines expressing IDO1 mutants, mimicking the phosphorylation of one or both ITIMs. As the mock control cells, P1.HTR cells were transfected with the empty plasmid. We first quantified *Ido1* transcripts in Y115E, Y253E and Y115_253E transfectants by real‐time PCR. Nucleotide changes did not affect the transcriptional activity of *Ido1* mutants, as compared to WT *Ido1* used as a calibrator (Fig. 2A). By immunoblotting WCLs obtained from stably transfected P1.HTR cells, we quantified the expression of mutated IDO1 at the post‐transcriptional level. Different from mock controls, transfection of either WT or mutated gene constructs led to detectable expression of a 42 kDa protein, matching the known size of mouse IDO1 (Fig. 2B). However, although comparable amounts of total proteins were loaded for each sample (as indicated by β‐tubulin expression), expression levels of ITIM‐mutated proteins appeared to be significantly lower relative to WT IDO1 (Fig. 2B), an effect substantiated by densitometric analysis (Fig. 2C).

We next examined the enzymatic activity of IDO1 mutants in terms of ability to secrete Kyn in culture supernatants after 6 or 16 hr of incubation with an extra amount of l‐Trp. Interestingly, both the singly and doubly mutated forms of IDO1 lost their catalytic activity as compared to WT controls (Table 2).

**Table 2:** Catalytic activity of ITIM‐mutated IDO1 transfectants

Sample	Kyn (μM) 6 hr	Kyn (μM) 16 hr
WT	5.825 ± 0.338	3.800 ± 0.212
Y115E	nd	nd
Y253E	nd	nd
Y115_253E	nd	nd

Kynurenine concentration, Kyn (μM), was assessed in the supernatants of untreated cells maintained 6 or 16 hr in culture in the presence of excess of Trp. The detection limit of the analysis was 0.05 μM. Concentrations below the detection limit are indicated as not detectable (nd). Data are means (±S.D.) of three experiments each performed in triplicate.

Thus, the characterization of IDO1 ITIM mutants in P1.HTR transfectants suggested that replacement of even an individual tyrosine with glutamate impairs protein but not transcript expression of IDO1, while causing complete loss of catalytic activity. Loss of catalytic function might be traced to a major change in the folding pattern of mutated *versus* WT IDO1 proteins, resulting from the introduction of a negative charge. As a matter of fact, the inspection of the human IDO1 structure (based on the available crystal structures), indicates a poor solvent exposure of tyrosine residues in ITIMs occurring in the small domain of the enzyme (Y115, Q_SASA_ = 0.083 ± 0.006; Y253, Q_SASA_ = 0.091 ± 0.007) (Fig. 2D). Therefore, ITIM phosphorylation likely results in major conformational changes in IDO1 and causes a significant change in shape of the catalytic cleft, affecting substrate‐binding and catalytic activity.

### Ability of ITIM‐mutated IDO1 to bind molecular partners

**Figure 3:**
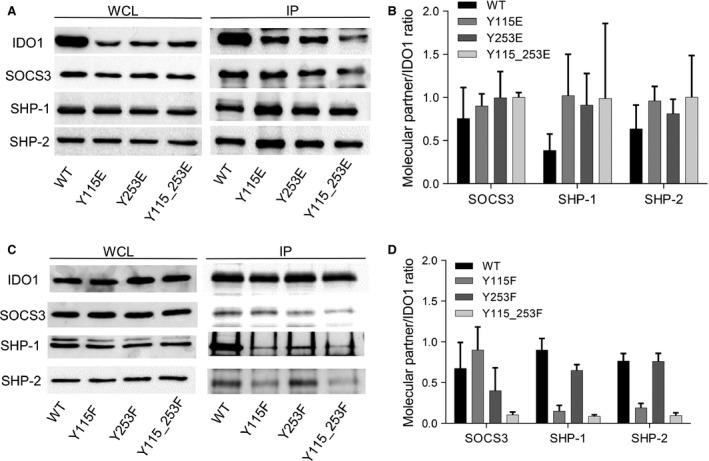
Association of ITIM‐mutated IDO1 with its molecular partners. (**A, C**) Immunoprecipitation (IP; right panel) of IDO1 from WCL of WT and ITIM‐mutated IDO1 transfectants, and detection of IDO1 or its molecular partners (SOCS3, SHP‐1 and SHP‐2) by sequential immunoblotting with specific antibodies. WCL (left panel), immunoblot analysis of whole‐cell lysates from stably transfected cells, as control of protein expression. One representative experiment is shown. (**B, D**) Quantitative analysis of triplicate gels (mean ± S.D.), one of which represented in (A, C). For each cell type, the ratio of co‐immunoprecipitated protein over immunoprecipitated IDO1 was evaluated.

In addition to its catalytic activity, IDO1 manifests a ‘protein moonlighting’ activity, a phenomenon whereby ancestral enzymes perform more than one function 8, 9. Phosphorylated ITIMs allow IDO1 to associate with different molecular partners responsible for different downstream effects 7, 8. Because the known molecular partners of IDO1 (*i.e*. SOCS3, SHP‐1 and SHP‐2) are constitutively expressed in P1.HTR cells and, differently from IDO1 expression, showed a comparable expression level in WT, Y115E, Y253E and Y115_253E transfectants (Fig. 3A, left panel), we analysed the ability of pITIM‐mimic mutants of IDO1 to bind those proteins. The same analysis was conducted on Y115F, Y253F and Y115_253F transfectants in which a phenylalanine (F) replaces the phosphorylable tyrosine of IDO1's ITIMs, abrogating a single or both docking sites of the enzyme (Fig. 3C) 7, 8. Using WCLs from the same transfectant cells in combination with IDO1‐specific antibody, we performed immunoprecipitation experiments to obtain IDO1 precipitates to be assayed by immunoblotting for their relative amounts of molecular partners (*i.e*. SOCS3, SHP‐1 and SHP‐2) associated with the enzyme (Fig. 3A and C right panels). Quantitative analysis of molecular partners (in terms of specific partner to IDO1 ratio) indicated that all the pITIM‐mimic mutants would associate with SOCS3, SHP‐1 and SHP‐2 to an extent similar to, or even higher than, WT IDO1. Single pITIM‐mimic mutants did not reveal any specific association with one of the molecular partners (Fig. 3B). As previously described, neither SOCS3 nor SHP‐1 and SHP‐2 could bind Y115_253F mutant, while the single Y115F and Y253F showed a specific tendency to associate SOCS3 and SHP phosphatases respectively (Fig. 3D).

Thus, IDO1 mutants mimicking the stable phosphorylation of one or two ITIMs maintain the ability to bind SOCS3, SHP‐1 and SHP‐2, although the single Y115E and Y253E could not discriminate between SOCS3 and SHP phosphatases.

### The ITIM2‐mutation controls the IDO1 protein stability

**Figure 4:**
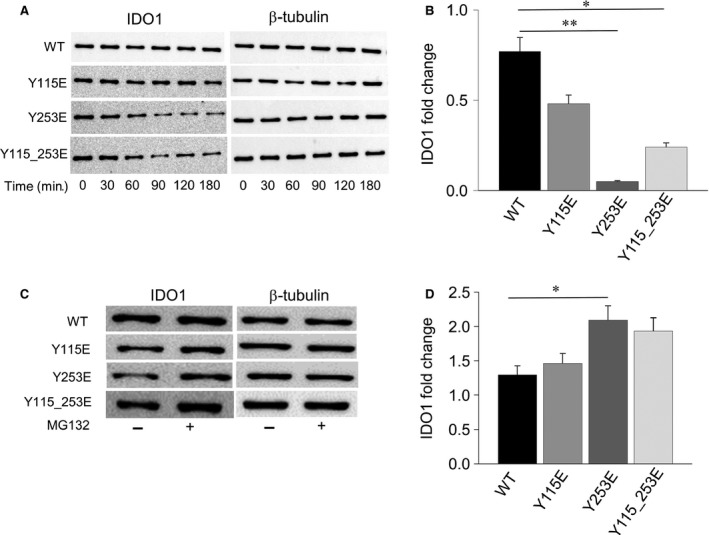
Half‐life of ITIM‐mutated IDO1 proteins. (**A**) Cycloheximide (50 μM) was added to the cultures and transfected P1.HTR tumour cells were harvested at the indicated time‐points (0–180 min.). WCL was subjected to immunoblot analysis, using an IDO1‐specific antibody. Detection of β‐tubulin served as a loading control. One representative experiment is shown. (**B**) Quantitative analysis of triplicate gels (mean ± S.D.), one of which represented in (A). For each sample, the ratio of normalized IDO1 protein at 180 min. over time 0 was represented (IDO1 fold change within 180 min.). *P < 0.05, **P < 0.01, mutants *versus* WT (Student’*s t*‐test). (**C**) Proteasome inhibition restores IDO1 expression in transfected cells. Cells were treated with MG132 (at 10 μM for 180 min.) before WCLs were collected for immunoblot analysis of IDO1, using β‐tubulin detection as a loading control. One representative experiment of three is shown. (**D**) Quantitative analysis of triplicate gels (mean ± S.D.), one of which represented in (C). For each cell type, the ratio of normalized IDO1 protein in MG132‐treated over untreated cells was evaluated by densitometry (IDO1 fold change upon proteasome inhibition). *P < 0.05, mutant *versus* WT (Student’*s t*‐test).

Although it has previously been shown that IDO1's half‐life depends on phosphorylated ITIMs 7, 8, the relative contribution of each ITIM has not been elucidated yet. We thus compared the protein half‐life of ITIM mutants with that of WT IDO1 over time. We subjected WT, Y115E, Y253E and Y115_253E transfected cells to treatment for 0–180 min. with the translational inhibitor cycloheximide, followed by Western blotting. All ITIM mutants, when compared to the WT protein, were characterized, in general, by a faster turnover (Fig. 4A). Interestingly, on measuring the fold change in β‐tubulin‐normalized IDO1 expression at 180 min., we found a highly significant decrease (*P* < 0.001), particularly in the expression of the Y253E mutant – mimicking tyrosine phosphorylation in ITIM2 – relative to WT IDO1. Although less significantly (*P* < 0.05), the protein fold change in the double Y115_253E mutant also decreased over time (Fig. 4B**)**.

To ascertain any involvement of proteasome activity in the accelerated turnover of proteins encoded by ITIM mutants, cells were treated with MG132, a proteasome inhibitor, for 180 min. Proteasome inhibition rescued IDO1 expression over the timeframe of observation in all of the stably transfected cell types (Fig. 4C**)**, indicating that the degradation of both WT and ITIM‐mutated IDO1 protein is indeed proteasome‐mediated. Moreover, quantitative analysis of IDO1 fold changes in MG132‐treated cells revealed a significant increase in the expression of the Y253E mutant (Fig. 4D), undergoing to the most rapid turnover among ITIM mutants (Fig. 4B). These results highlight a scenario whereby phosphorylation of only one of IDO1's ITIMs – namely, ITIM2 – presides over IDO1 protein stability.

### The ITIM1‐mutation increases the phosphatase activity associated with IDO1 protein

**Figure 5:**
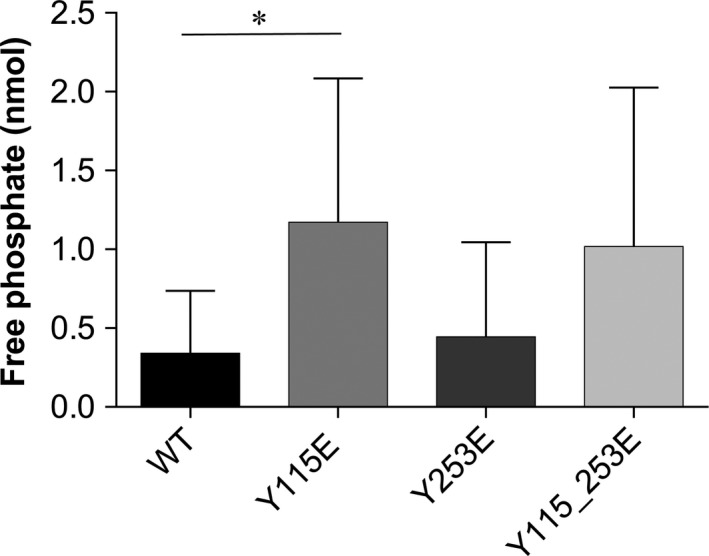
Phosphatase activity of ITIM‐mutated IDO1. Levels of free phosphate generated during the incubation of a tyrosine‐phosphorylated peptide with IDO1 proteins immunoprecipitated with an IDO1‐specific antibody from each WT or ITIM‐mutated IDO1 transfectants, according to the manufacturer's procedure (Sigma‐Aldrich). Results are representative of four experiments (mean ± S.D.). *P < 0.05, mutant *versus* WT (Student’*s t*‐test).

Besides SOCS3, also SHP‐1 and SHP‐2 phosphatases associate with IDO1 through phosphorylated ITIMs, an event that initiates a signalling function by the enzyme 8, 13. We thus investigated any associated phosphatase activity of immunoprecipitated IDO1 mutants in terms of an ability to remove a phosphate group from tyrosine‐phosphorylated substrate. The immunoprecipitate‐associated phosphatase activity was significantly increased in cells expressing the Y115E mutant, whereas co‐immunoprecipitation experiments of cells expressing Y253E generated a level of free phosphate similar to WT IDO1. Although the phosphatase activity associated with the double Y115_253E mutant showed a tendency towards increased activity relative to the WT protein, the change was not statistically significant (Fig. 5). Therefore, unlike ITIM2‐mutated IDO1, responsible for proteasomal degradation of the enzyme (Fig. 4), the ITIM1‐mutated counterpart appeared to be crucial in recruiting a phosphatase entity to IDO1, further substantiating the hypothesis of differential roles for the two phosphorylated forms of IDO1.

### ITIM1‐mutation triggers IDO1 signalling in pDCs

**Figure 6:**
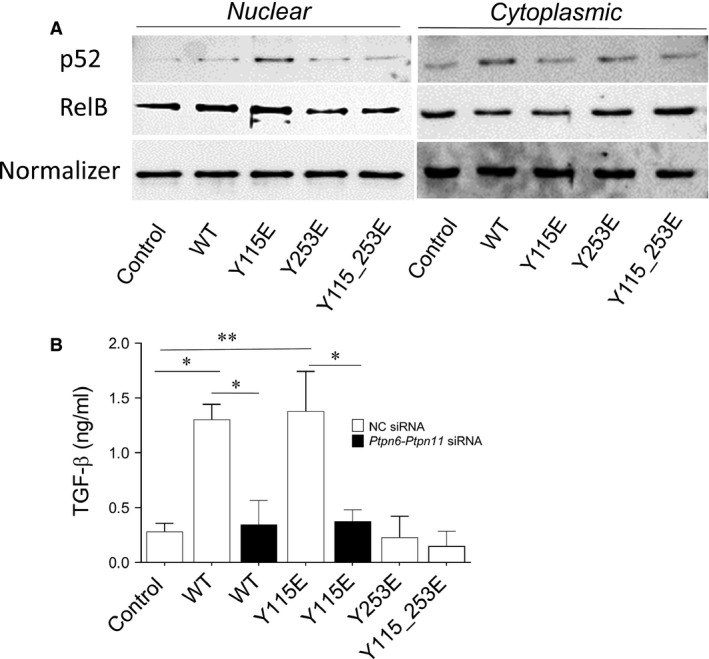
Activation of the noncanonical pathway of NF‐κB and TGF‐β production in *Ido1* ^*−/−*^ pDCs transfected with ITIM‐mutants. (**A**) Expression of p52 and RelB in nuclear and cytoplasmic extracts from *Ido1* ^*−/−*^ pDCs transfected with the WT and ITIM‐mutated forms of *Ido1*. The control was represented by *Ido1* ^*−/−*^ pDCs transfected with a control mRNA. Lamin B1 and β‐tubulin were used as loading control of nuclear and cytoplasmic extracts respectively. One representative experiment is shown. (**B**) TGF‐β production in the supernatants of *Ido1* ^*−/−*^ pDCs transfected with the WT and ITIM‐mutated forms of *Ido1*. WT and Y115E‐transfected *Ido1* ^*−/−*^ pDCs were gene silenced by mixed siRNAs targeting *Ptnp6* and *Ptnp11*. Results are representative of three experiments (mean ± S.D.). *P < 0.05, **P < 0.01 (experimental *versus* control sample; Student’*s t*‐test).

The IDO1‐SHP axis activates intracellular signalling events in pDCs, mediated by the non‐canonical NF‐κB pathway. This requires TGF‐β production, responsible for the acquisition of a durable immunoregulatory phenotype in pDCs 8, 9. The molecular events downstream of ITIM phosphorylation in IDO1 – namely, activation of phosphatase activity by SHPs – involve nuclear translocation of the noncanonical p52 and RelB subunits of NF‐κB, and they culminate in the production of TGF‐β and induction of a regulatory phenotype in pDCs. To gain insight into the signalling events initiated by the Y115E mutant, we evaluated nuclear translocation of p52 and RelB subunits and TGF‐β production in pDCs transfected with ITIM‐mutated *Ido1*. To rule out any signalling effect triggered by endogenous IDO1 expressed by wild‐type pDCs, ITIM mutants were transfected into *Ido1* ^−/−^ pDCs. Analysis of the activation pattern of non‐canonical NF‐κB revealed an increased expression of p52 and RelB in normalized nuclear extracts of cells transfected with the Y115E mutant, with a minor increase in p52 nuclear translocation in IDO1 WT transfectants, as compared to control. In contrast, no significant translocation of p52 and RelB was observed in Y253E and Y115_253E‐transfected pDCs (Fig. 6A, left panel). A decreased level of p52 and RelB was observed in normalized cytoplasmic extract of Y115E mutant (Fig. 6A, right panel), confirming the nuclear translocation of the non‐canonical subunits of NF‐κB.

Since TGF‐β represents one of the downstream effectors of the signalling activity of IDO1 and it is responsible for a long‐term immunoregulatory phenotype of pDC 8, 30, we next determined the cytokine secretion in supernatants from transfected pDCs. Cytokine levels appeared significantly and specifically increased in WT‐ and ITIM1‐transfected cells, as compared to controls, whereas single ITIM2‐ or double ITIM1_2‐transfected pDCs produced a level of TGF‐β similar to controls. Phosphatase activity of SHP‐1 and SHP‐2 was required for the production of the cytokine by WT‐ and ITIM1‐transfected pDCs, since the combined silencing of *Ptpn6*‐*Ptpn11* (coding for SHP‐1 and SHP‐2 respectively) significantly decreased TGF‐β level (Fig. 6B). The selective activation of a signalling involving non‐canonical NF‐κB pathway and TGF‐β production by ITIM1‐tranfected *Ido1* ^−/−^ pDCs – but neither by ITIM2‐ nor ITIM1_2‐transfected pDCs – suggested a specific role for phosphorylated ITIM1, rather than ITIM2, in mediating the signalling activity of IDO1 in the pDCs.

### ITIM1‐mediated signalling confers immunosuppressive properties on pDCs detectable *in vivo*

**Figure 7:**
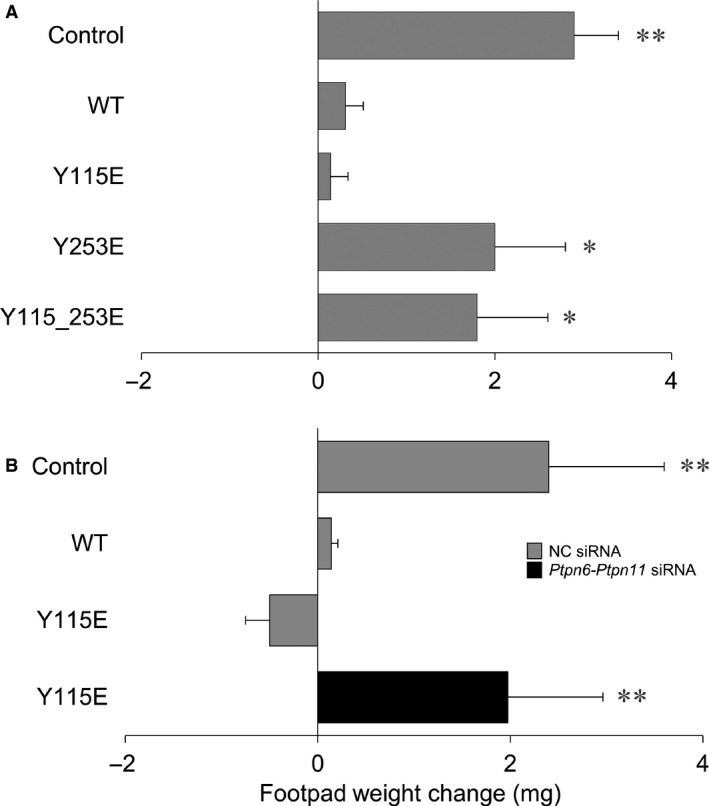
Skin test reactivity of mice immunized with pDCs transfected with ITIM‐mutated *Ido1*. (**A**) Splenic HY‐pulsed cDCs in combination with a minority fraction (5%) of *Ido1* ^*−/−*^ pDCs were transferred into syngeneic C57BL/6 recipient mice to be assayed for skin reactivity to the eliciting peptide. Before the antigen pulsing, pDCs were transfected with the WT or ITIM‐mutated forms of *Ido1*. The control was represented by *Ido1* ^*−/−*^ pDCs transfected with a control mRNA. (**B**) ITIM1‐(Y115E)‐transfected *Ido1* ^*−/−*^ pDCs were gene silenced by mixed siRNAs targeting *Ptnp6* and *Ptnp11* and used in combination with cDCs for skin reactivity. The control was represented by negative control (NC) siRNA‐transfected *Ido1* ^*−/−*^ pDCs. Analysis of skin reactivity of recipient mice (*n* = 6 per group) to the eliciting peptide is presented as change in footpad weight. Results are representative of three experiments (mean ± S.D.). *P < 0.05, **P < 0.01 (experimental *versus* control footpads; two‐tailed paired *t*‐test).

To further characterize the biological relevance of the signalling activity of the ITIM1 mutant, we evaluated the *in vivo* priming ability of *Ido1* ^*−/−*^ pDCs transfected with the WT or either ITIM‐mutated forms of IDO1. We sensitized female mice with the *H‐2D* ^b^–restricted HY peptide presented by a combination of immunogenic cDCs and a minority fraction of transfected *Ido1* ^*−/−*^ pDCs. Two weeks later, we evaluated reactivity to intrafootpad challenge with the HY antigen, according to an established protocol for measuring the induction of immunoreactivity *versus* tolerance 22, 26, 31, 32, 33. As expected, overexpression of WT IDO1 elicited an immunosuppressive phenotype in *Ido1* ^−/−^ pDCs, such that antigen sensitization by the otherwise immunogenic DCs was negated 13. Interestingly, we found that Y115E was the only ITIM‐mutant capable of providing *Ido1* ^−/−^ pDCs with the same immunosuppressive functional phenotype as that of WT IDO1‐transfected cells. As a matter of fact, both Y253E‐ and Y115_253E‐transfected pDCs failed to activate an antigen‐specific immunosuppressive response in recipient mice (Fig. 7A). Because all three types of IDO1 mutant are catalytically inactive and the pDCs were deficient in endogenous *Ido1* expression, these data suggest that the immunosuppressive effect of Y115E is attributable solely to IDO1's signalling activity.

To assess the association between the signalling activity of the ITIM1 mutant and the acquisition of a regulatory phenotype by pDCs, the priming ability of ITIM1‐transfected pDCs was evaluated after pre‐treatment with siRNAs targeting *Ptpn6*‐*Ptpn11*. We observed that both the individual knockdown of either phosphatase (data not shown) and the combined silencing of SHP‐1/SHP‐2 would negate the immunosuppressive phenotype of ITIM1‐transfected pDCs (Fig. 7B), suggesting that the immunosuppressive function conferred on *Ido1* ^*−/−*^ pDCs by Y115E − but neither by Y253E nor by Y115_253E − is mediated by the specific ability of the IDO1 mutant to bind catalytically active SHP1/2 phosphatases.

## Discussion

Immunoreceptor tyrosine‐based inhibitory motif sequences are evolutionarily conserved regulatory motifs 34. In addition to its catalytic activity, ITIM‐related functions provide IDO1 with an ability to either up‐ or down‐regulate its own expression in DCs. Under pro‐inflammatory conditions dominated by IL‐6, pIDO1 associates with SOCS3, thus accelerating the enzyme's turnover and terminating its functioning 7. SOCS3, once associated with a tyrosine‐phosphorylated protein *via* its SH2 domain, will indeed recruit the E3 ubiquitin ligase complex through its SOCS box domain and target any associated proteins, including IDO1, for proteasomal degradation 35, 36.

However, in a different cytokine milieu, tyrosine phosphorylation can provide IDO1's ITIMs with an ability to interact with other SH2‐containing proteins – namely, phosphatases – thus, triggering downstream signalling effects 8. This evolutionarily selected mechanism allows proteins characterized by pre‐existing functions (*i.e*. receptorial and/or enzymatic) to engage in novel activities, to meet any newly emerged environmental needs. Of note, solvent exposure of the phosphorylation sites – namely, Y115 and Y253 – is very low in all of the available crystal structures of IDO1 (solvent accessible surface area <0.5 Å^2^), confirming that major conformational changes may occur in the non‐catalytic small domain in specific microenvironment conditions, so to favour exposure and/or selective phosphorylation of ITIMs.

Because the dynamic identification of distinct phosphorylated forms of IDO1 under specific conditions could be difficult to observe in living cells, owing in part to the short half‐life of phospho‐(Y)‐ITIMs, we reasoned that an alternative approach could be the use of stable recombinant phospho‐(Y)‐mimetic IDO1 mutants, *via* replacement of phosphorylable tyrosines with glutamate residues 27, 28, 29. Mimicking the negative charge of the tyrosine‐bound phosphate group, glutamate allows the stabilization of a folding conformation similar to that obtained in the presence of tyrosine phosphorylation. Although different from physiological tyrosine phosphorylation, IDO1 mutants containing one or two glutamate residues could be expected to mimic stable and specific phosphorylation of their ITIMs.

Transcript expression of those mutants did not differ from that of WT *Ido1* in transfected cells. However, despite detectable translation into IDO1 protein (which was, however, lower than in the WT control), we observed complete loss of Trp‐degrading activity in cells transfected with all mutants. These data therefore suggest that the negative charge(s) carried by the glutamate residue(s) – or, physiologically, by phosphorylated tyrosine(s) – greatly affects IDO1's folding conformation and thus modify the shape of the catalytic site. This also suggests that ITIM phosphorylation occurs within an IDO1 conformation different from that hosting a ligand at the catalytic site. A folding conformation not compatible with the one required for the signalling function of IDO1 might be required for the enzymatically active form of IDO1, implying that the catalytic and ITIM‐dependent activities of IDO1 are not only functionally segregated but also mutually exclusive, as dictated by the local environmental needs. The available crystal structures of ligand‐bound IDO1 point indeed to ITIMs with poor solvent exposures of Y115 and Y253, which are not amenable to post‐transcriptional modifications, including phosphorylation.

In spite of the loss of catalytic activity, the IDO1 mutants maintained their ability to associate with SOCS3 or SHP‐1/SHP‐2 phosphatases, allowing triggering of ITIM‐related functions. We observed a faster dropdown of Y253E and Y115_253E relative to Y115E and more so WT proteins, which was mediated by a proteasomal mechanism. These results highlight a pivotal role of Y253E, mimicking pITIM2, in the proteasome‐mediated degradation of IDO1, in spite of the lack of a specific binding ability in associating SOCS3. To further clarify the apparently disparate functions of phosphorylated IDO1's ITIMs, we analysed the signalling activity resulting from the association of the mutants with the tyrosine protein phosphatases, SHP1 and SHP2. The phosphatase activity co‐immunoprecipitated with the different forms of IDO1 was significantly increased in Y115E, mimicking pITIM1, and it was lost in the other mutants that can associate with SHP1/2 phosphatases yet. Although all the pITIM‐mimic mutants could associate whichever molecular partners of IDO1, the resulting ITIM‐dependent activities of the enzyme – namely, proteasome degradation *via* SOCS3 and molecular signalling *via* SHPs – are associated with distinct phospho‐mimetic IDO1 mutants. This observation confirmed the non‐overlapping function of the two ITIMs and the requirement of a cellular milieu for switching on a specific ITIM function. Although the double mutant could be expected to behave as the single ITIM1 mutant, these data suggested that the copresence of a negatively charged ITIM2 might either confer a distinct conformation on IDO1 – making it incapable of transducing signals – or divert IDO1 functioning and/or drive the direct proteasomal degradation of the enzyme. Phosphorylation of either ITIM1 or ITIM2 will thus provide the enzyme with a different ITIM‐related fate/function, depending on the needs of the local microenvironment. Specifically, the ITIM‐mediated IDO1 plasticity would translate in DCs into opposite functional phenotypes, that is*,* immunoadjuvant *versus* immunoregulatory in nature 9. In this regard, it should be noted that pDCs represent the most flexible DC subset, capable of rapidly shifting from an immunostimulatory to an immunosuppressive programme and *vice versa* in response to inflammatory *versus* anti‐inflammatory signals 25, 37. The inflammatory *versus* anti‐inflammatory milieu sensed by pDC may promote not only a differential expression of the IDO1's molecular partner (*i.e*. SOCS3 *versus* SHP1/2 phosphatases) 9 but also it should activate distinct tyrosine kinases that exclusively phosphorylate just one of the two IDO1's ITIM, according to a general mechanism of phosphorylation of dual ITIM‐containing receptors. In a similar fashion, the prototypical ITIM‐bearing receptor FcγRIIB contains two distinct ITIMs that can recruit different molecular partners, such as SHIP phosphatases and the adapters Grb2 and Grap 38. A mechanism of phosphorylation of dual ITIM‐containing receptors has been proposed for Siglec‐9 and PECAM‐1, involving a sequential phosphorylation of the two ITIM motifs operated by different *Src* tyrosine kinases 39. Several examples of dual/multiple ITIM‐containing receptors exist where consecutive phosphorylation occurs for generating sequential docking sites anchoring different intermediates that convey in a unique outcome, including the transduction of the inhibitory signaling.

The identification of distinct roles for the two pITIMs of IDO1 may pave the way to innovative strategies in treating autoimmune, chronic inflammatory and neoplastic diseases by either enhancing or terminating IDO1's activity, as appropriate. The finding that the enzymic and signalling functions of IDO1 appear to be mutually exclusive is important in that it may be key to a better understanding of dysfunctional IDO1‐dependent regulation of immunity *versus* tolerance in several disease states 40, 41. Interventions on IDO1 signaling function may provide further therapeutic opportunities to make the protein an effectively druggable target. In conclusion, the identification of IDO1 as a dual ITIM protein, that can activate independent molecular mechanisms leading to different outcome, offers new opportunities for targeting IDO1 through novel strategies in addition to the mere inhibition of its catalytic function.

## Conflict of interest

The authors declare that they have no conflicts of interest with the contents of this article.

## Author contributions

CO designed the study and wrote the paper. UG and PP supervised the experiments. MG, MLB and FF cloned IDO1 mutants. EA,VR, MTP, CA and ClVo designed and performed *in vitro* experiments and analysed the data. GM, RB and CaVa performed *in vivo* experiments. AM performed computational analysis of IDO1's ITIMs. All authors reviewed results and approved the final version of the manuscript.

## References

[1] Platten M , von Knebel Doeberitz N , Oezen I , *et al* Cancer immunotherapy by targeting IDO1/TDO and their downstream effectors. Front Immunol. 2014; 5: 673. 25628622 10.3389/fimmu.2014.00673 PMC4290671

[2] Fallarino F , Grohmann U , Puccetti P . Indoleamine 2,3‐dioxygenase: from catalyst to signaling function. Eur J Immunol. 2012; 42: 1932–7. 22865044 10.1002/eji.201242572

[3] Fallarino F , Grohmann U . Using an ancient tool for igniting and propagating immune tolerance: IDO as an inducer and amplifier of regulatory T cell functions. Curr Med Chem. 2011; 18: 2215–21. 21517758 10.2174/092986711795656027

[4] Munn DH , Mellor AL . Indoleamine 2,3 dioxygenase and metabolic control of immune responses. Trends Immunol. 2013; 34: 137–43. 23103127 10.1016/j.it.2012.10.001 PMC3594632

[5] Belladonna ML , Puccetti P , Orabona C , *et al* Immunosuppression via tryptophan catabolism: the role of kynurenine pathway enzymes. Transplantation. 2007; 84: S17–20. 10.1097/01.tp.0000269199.16209.22 17632406

[6] Belladonna ML , Volpi C , Bianchi R , *et al* Cutting edge: autocrine TGF‐beta sustains default tolerogenesis by IDO‐competent dendritic cells. J Immunol. 2008; 181: 5194–8. 18832670 10.4049/jimmunol.181.8.5194

[7] Orabona C , Pallotta MT , Volpi C , *et al* SOCS3 drives proteasomal degradation of indoleamine 2,3‐dioxygenase (IDO) and antagonizes IDO‐dependent tolerogenesis. Proc Natl Acad Sci USA. 2008; 105: 20828–33. 19088199 10.1073/pnas.0810278105 PMC2634889

[8] Pallotta MT , Orabona C , Volpi C , *et al* Indoleamine 2,3‐dioxygenase is a signaling protein in long‐term tolerance by dendritic cells. Nat Immunol. 2011; 12: 870–8. 21804557 10.1038/ni.2077

[9] Orabona C , Pallotta MT , Grohmann U . Different partners, opposite outcomes: a new perspective of the immunobiology of indoleamine 2,3‐dioxygenase. Mol Med. 2012; 18: 834–42. 22481272 10.2119/molmed.2012.00029 PMC3409287

[10] Ravetch JV , Lanier LL . Immune inhibitory receptors. Science. 2000; 290: 84–9. 11021804 10.1126/science.290.5489.84

[11] Daeron M , Jaeger S , Du Pasquier L , *et al* Immunoreceptor tyrosine‐based inhibition motifs: a quest in the past and future. Immunol Rev. 2008; 224: 11–43. 18759918 10.1111/j.1600-065X.2008.00666.x

[12] Long EO . Negative signaling by inhibitory receptors: the NK cell paradigm. Immunol Rev. 2008; 224: 70–84. 18759921 10.1111/j.1600-065X.2008.00660.x PMC2587243

[13] Pallotta MT , Orabona C , Bianchi R , *et al* Forced IDO1 expression in dendritic cells restores immunoregulatory signalling in autoimmune diabetes. J Cell Mol Med. 2014; 18: 2082–91. 25215657 10.1111/jcmm.12360 PMC4193887

[14] Fallarino F , Uyttenhove C , Boon T , *et al* Endogenous IL‐12 is necessary for rejection of P815 tumor variants in vivo. J Immunol. 1996; 156: 1095–100. 8557984

[15] Berman HM , Westbrook J , Feng Z , *et al* The protein data bank. Nucleic Acids Res. 2000; 28: 235–42. 10592235 10.1093/nar/28.1.235 PMC102472

[16] Sugimoto H , Oda S , Otsuki T , *et al* Crystal structure of human indoleamine 2,3‐dioxygenase: catalytic mechanism of O2 incorporation by a heme‐containing dioxygenase. Proc Natl Acad Sci USA. 2006; 103: 2611–6. 16477023 10.1073/pnas.0508996103 PMC1413787

[17] Tojo S , Kohno T , Tanaka T , *et al* Crystal structures and structure‐activity relationships of imidazothiazole derivatives as IDO1 inhibitors. ACS Med Chem Lett. 2014; 5: 1119–23. 25313323 10.1021/ml500247w PMC4190630

[18] Peng YH , Ueng SH , Tseng CT , *et al* Important hydrogen bond networks in indoleamine 2,3‐dioxygenase 1 (IDO1) sith IDO1. J Med Chem. 2016; 59: 282–93. 26642377 10.1021/acs.jmedchem.5b01390

[19] Cavallo L , Kleinjung J , Fraternali F . POPS: A fast algorithm for solvent accessible surface areas at atomic and residue level. Nucleic Acids Res. 2003; 31: 3364–6. 12824328 10.1093/nar/gkg601 PMC169007

[20] Mizushima S , Nagata S . pEF‐BOS, a powerful mammalian expression vector. Nucleic Acids Res. 1990; 18: 5322. 1698283 10.1093/nar/18.17.5322 PMC332193

[21] Romani L , Fallarino F , De Luca A , *et al* Defective tryptophan catabolism underlies inflammation in mouse chronic granulomatous disease. Nature. 2008; 451: 211–5. 18185592 10.1038/nature06471

[22] Grohmann U , Fallarino F , Bianchi R , *et al* A defect in tryptophan catabolism impairs tolerance in nonobese diabetic mice. J Exp Med. 2003; 198: 153–60. 12835483 10.1084/jem.20030633 PMC2196078

[23] Grohmann U , Orabona C , Fallarino F , *et al* CTLA‐4‐Ig regulates tryptophan catabolism in vivo. Nat Immunol. 2002; 3: 1097–101. 12368911 10.1038/ni846

[24] Orabona C , Grohmann U , Belladonna ML , *et al* CD28 induces immunostimulatory signals in dendritic cells via CD80 and CD86. Nat Immunol. 2004; 5: 1134–42. 15467723 10.1038/ni1124

[25] Grohmann U , Volpi C , Fallarino F , *et al* Reverse signaling through GITR ligand enables dexamethasone to activate IDO in allergy. Nat Med. 2007; 13: 579–86. 17417651 10.1038/nm1563

[26] Grohmann U , Bianchi R , Orabona C , *et al* Functional plasticity of dendritic cell subsets as mediated by CD40 versus B7 activation. J Immunol. 2003; 171: 2581–7. 12928409 10.4049/jimmunol.171.5.2581

[27] Rudel S , Wang Y , Lenobel R , *et al* Phosphorylation of human Argonaute proteins affects small RNA binding. Nucleic Acids Res. 2011; 39: 2330–43. 21071408 10.1093/nar/gkq1032 PMC3064767

[28] Tomar A , Lawson C , Ghassemian M , *et al* Cortactin as a target for FAK in the regulation of focal adhesion dynamics. PLoS ONE. 2012; 7: e44041. 22952866 10.1371/journal.pone.0044041 PMC3430618

[29] Stateva SR , Salas V , Benaim G , *et al* Characterization of phospho‐(tyrosine)‐mimetic calmodulin mutants. PLoS ONE. 2015; 10: e0120798. 25830911 10.1371/journal.pone.0120798 PMC4382182

[30] Chen W . IDO: more than an enzyme. Nat Immunol. 2011; 12: 809–11. 21852775 10.1038/ni.2088

[31] Orabona C , Belladonna ML , Vacca C , *et al* Cutting edge: silencing suppressor of cytokine signaling 3 expression in dendritic cells turns CD28‐Ig from immune adjuvant to suppressant. J Immunol. 2005; 174: 6582–6. 15905495 10.4049/jimmunol.174.11.6582

[32] Orabona C , Tomasello E , Fallarino F , *et al* Enhanced tryptophan catabolism in the absence of the molecular adapter DAP12. Eur J Immunol. 2005; 35: 3111–8. 16206234 10.1002/eji.200535289

[33] Orabona C , Puccetti P , Vacca C , *et al* Toward the identification of a tolerogenic signature in IDO‐competent dendritic cells. Blood. 2006; 107: 2846–54. 16339401 10.1182/blood-2005-10-4077

[34] Abu‐Dayyeh I , Ralph B , Grayfer L , *et al* Identification of key cytosolic kinases containing evolutionarily conserved kinase tyrosine‐based inhibitory motifs (KTIMs). Dev Comp Immunol. 2010; 34: 481–4. 20043942 10.1016/j.dci.2009.12.012

[35] Orr SJ , Morgan NM , Buick RJ , *et al* SOCS3 targets Siglec 7 for proteasomal degradation and blocks Siglec 7‐mediated responses. J Biol Chem. 2007; 282: 3418–22. 17138568 10.1074/jbc.C600216200

[36] Orr SJ , Morgan NM , Elliott J , *et al* CD33 responses are blocked by SOCS3 through accelerated proteasomal‐mediated turnover. Blood. 2007; 109: 1061–8. 17008544 10.1182/blood-2006-05-023556

[37] Volpi C , Fallarino F , Pallotta MT , *et al* High doses of CpG oligodeoxynucleotides stimulate a tolerogenic TLR9‐TRIF pathway. Nat Commun. 2013; 4: 1852. 23673637 10.1038/ncomms2874

[38] Isnardi I , Lesourne R , Bruhns P , *et al* Two distinct tyrosine‐based motifs enable the inhibitory receptor FcgammaRIIB to cooperatively recruit the inositol phosphatases SHIP1/2 and the adapters Grb2/Grap. J Biol Chem. 2004; 279: 51931–8. 15456754 10.1074/jbc.M410261200

[39] Tourdot BE , Brenner MK , Keough KC , *et al* Immunoreceptor tyrosine‐based inhibitory motif (ITIM)‐mediated inhibitory signaling is regulated by sequential phosphorylation mediated by distinct nonreceptor tyrosine kinases: a case study involving PECAM‐1. Biochemistry. 2013; 52: 2597–608. 23418871 10.1021/bi301461t PMC3666314

[40] Maghzal GJ , Winter S , Wurzer B , *et al* Tryptophan catabolism is unaffected in chronic granulomatous disease. Nature. 2014; 514: E16–7. 25341792 10.1038/nature13844

[41] Romani L , Puccetti P . Romani & Puccetti reply. Nature. 2014; 514: E18. 25341793 10.1038/nature13845

